# Case report: A case of new mutation in *SERPINC1* leading to thrombotic microangiopathy

**DOI:** 10.3389/fgene.2023.1278511

**Published:** 2023-09-27

**Authors:** Bing Li, Xiaohui Zhang, Hailin Lv, Xiaoqing Yang, Yanxia Gao, Zhao Hu, Chengjun Ma

**Affiliations:** ^1^ Department of Nephrology, Cheeloo College of Medicine, Qilu Hospital, Shandong University, Qingdao, China; ^2^ Department of Pathology, The First Affiliated Hospital of Shandong First Medical University, Jinan, China; ^3^ Department of Nephrology, Cheeloo College of Medicine, Qilu Hospital, Shandong University, Jinan, China

**Keywords:** hereditary antithrombin III deficiency, thrombotic microangiopathy, SERPINC1 gene mutation, thrombosis, case report

## Abstract

**Introduction:** Hereditary antithrombin-III deficiency can significantly increase the risk for thrombosis, which is common in limb deep vein and pulmonary cases. However, thrombotic microangiopathy (TMA) caused by hereditary antithrombin deficiency is rare.

**Case Presentation:** We reported the case of a 32-year-old Chinese female patient with TMA with renal injury caused by decreased antithrombin-III activity due to a new mutation (chr1-173884049 c.50A>G) in *SERPINC1*, which encodes antithrombin-III. In this case, the patient had no history of relevant drug use, diabetes, or monoclonal plasma cells in the bone marrow puncture. Consequently, TMA of the kidney was considered secondary to hereditary antithrombin-III deficiency. Gene detection was the only clue that led us to suspect that TMA was caused by hereditary antithrombin deficiency.

**Conclusion:** Our findings indicated that for patients with repeated findings of antithrombin-III activity less than 50%, the possibility of antithrombin-III deficiency and complete gene detection must be considered immediately after excluding the use of anticoagulants and lack of availability to facilitate early detection, diagnosis, and intervention.

## Introduction

Thrombotic microangiopathy (TMA) is characterized by microangiopathic hemolytic anemia, thrombocytopenia, and organ damage. Its pathological features include endothelial cell lesions in small arteries and capillaries, with thrombosis in the lumen of some small blood vessels (George and Nester, 2014). TMA can be divided into hemolytic uremic syndrome (HUS), thrombotic thrombocytopenic purpura (TTP), and secondary TMAs, and its etiology needs to be determined through histopathology and clinical and laboratory examinations (Brocklebank et al., 2018). Other researchers have proposed that secondary factors, such as drugs, autoimmune diseases, pregnancy, infection, tumors, organ transplantation, glomerulonephritis, and other diseases can trigger TMA under specific conditions. Based on the pathogenesis, some of them can be classified into HUS or TTP; however, not all secondary TMAs have clear etiology and pathogenesis. Thus, we propose to classify them as “TMA caused by other factors” (Claes et al., 2018).

Antithrombin-III is the primary anticoagulant used in the body, which inhibits thrombin and coagulation factors, such as II a, Ⅸ a, X a, XI a, and XII a (Kubier and O'Brien, 2012). Antithrombin-III, belonging to SERPIN superfamily of proteins, is a glycoprotein with a relative molecular weight of 58200 secreted by the liver and vascular endothelial cells. It comprises 432 amino acids and contains four glycosylation sites: Asn128, Asn167, Asn187, and Asn22. Antithrombin is encoded by *SERPINC1*, which is located on chromosome 1q23-25, with a total length of 13.4 kb. It consists of seven exons and six introns and contains two important structural and functional regions, which are the heparin binding site region located at the N-terminus and the serine proteinase binding region located at the C-terminus (Bravo-PerezVicente et al., 2019). Under physiological conditions, the activity of antithrombin is low, whereas heparin can increase its anticoagulant activity by up to 1000 times (Olson et al., 2010). Hereditary antithrombin-III deficiency is often associated with venous thromboembolic events (Di Minno et al., 2015); however, TMA is less common. This study reports a case of TMA with renal injury probably caused by decreased antithrombin-III activity due to a new mutation in *SERPINC1*, which encodes antithrombin-III.

## Case description

A 32-year-old Chinese woman with proteinuria for 3 years and creatinine rising to 151 μmol/L for 2 years was admitted to the nephrology department. One year prior to her visit to our hospital, a renal biopsy was performed at another hospital, which indicated mesangial dissolution. At that time, the assay showed creatinine at 151 μmol/L, anemia with hemoglobin at 69 g/L, and thrombocyte at 85 × 10^9^/L, and computed tomography (CT) showed no obvious cirrhosis. She was therefore prescribed with prednisone 30 mg once daily, and her creatinine fluctuated between 130 and 160 μmol/L. However, her anemia became worse. Twenty days before admission, the patient’s hemoglobin was 38 g/L and albumin was 27.3 g/L.

The patient had repeatedly undergone surgery for fundus vein thrombosis and rupture 3 years before admission. She had irregular menstruation owing to polycystic ovary syndrome.

**FIGURE 1:**
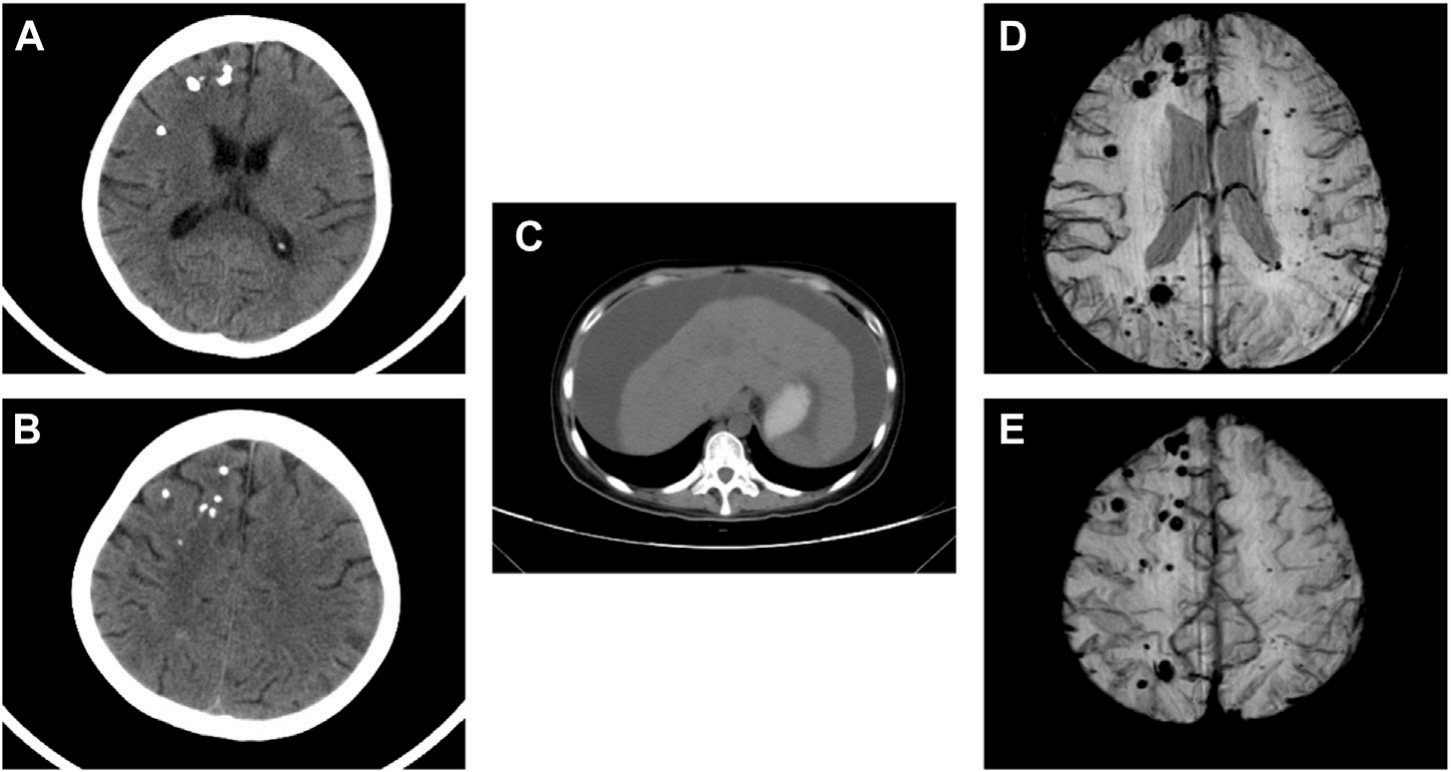
CT scan revealed multiple calcifications in the brain **(A,B)**. CT scan revealed cirrhosis and ascites **(C)**. Intracranial SWI showed multiple micro hemorrhage foci in addition to calcifications in the brain **(D,E)**. CT, computed tomography; SWI, susceptibility weighted imaging.

**FIGURE 2:**
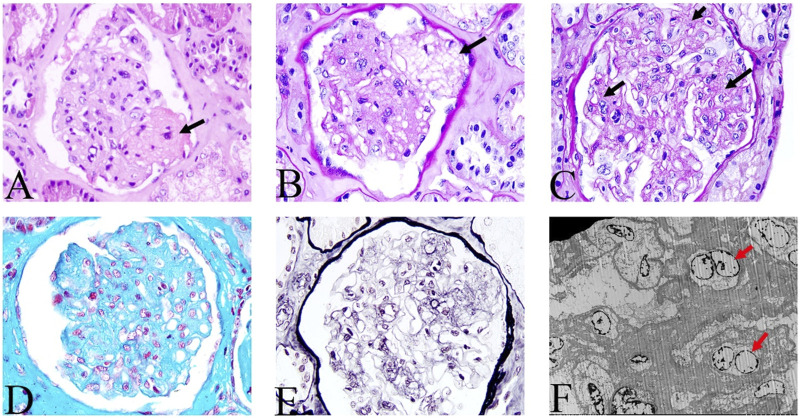
Histologic features of renal lesions. Mesangial lysis indicated by the black arrow. **(A)** hematoxylin and eosin; **(B,C)** periodic acid-Schiff; **(D)** Masson’s trichrome; **(E)** periodic acid-silver methenamine; (**A–E**, magnification, ×200). **(F)** Electron microscopic view of endothelial cells that are proliferating and swelling in pairs (as indicated by the red arrow), and no obvious electron compact deposition in the basement membrane and mesangial regions.

Physical examination after admission indicated that she was in a dystrophic state, with a hormonal face, sparse white hair, and normotension. She tested positive for ascites, but had no typical skin manifestations of liver disease such as palm erythema and spider angiomas. At presentation, the laboratory results revealed anemia and mildly elevated serum creatinine. The coagulation index showed that D-dimer was significantly increased up to 86.70 μg/mL and antithrombin-Ⅲ activity was 74%. The serological results on the admission day are shown in Supplementary Table S1. No abnormalities were found in the microbial, tumor, or rheumatic immune test results. CT revealed multiple calcifications in the brain (Figures 1A, B), cirrhosis, ascites, and pelvic effusion (Figure 1C). Intracranial susceptibility weighted imaging revealed multiple microhemorrhage foci in addition to calcifications in the brain (Figures 1D, E). Ultrasonography revealed left peroneal vein thrombosis. Histopathological images of previous renal punctures were reviewed and analyzed. The renal puncture biopsy tissue contained 14 glomeruli, four of which were spherically sclerotic, while the rest were diffuse mesangial lyses, showing diffuse proliferation and swelling of endothelial cells, shrinkage of capillary loops, and stenosis of the capillary lumen (Figure 2). Masson’s trichrome staining did not reveal immune complex deposition. The renal tubules were atrophied in sheets. Immunohistochemical analysis revealed negative results for immunoglobulin (Ig) G, IgA, IgM, IgM, C3, C1q, and kappa and lambda light chains along the capillary wall. Congo red staining results were negative. Electron microscopy revealed that the endothelial cells proliferated and swelled in pairs; and a wide, loose, and transparent area was observed between the subcutaneous and basement membranes. No obvious electron-compact deposition was observed in the basement membrane or mesangial region. Glomerular mesangial lysis is a mesangial injury caused by various harmful factors, which is common in drug toxicity, TMA, diabetic nephropathy, monoclonal immunoglobulin deposition, etc (Morita et al., 1998). In this case, the patient had no history of relevant drug use, diabetes, or monoclonal plasma cells in the bone marrow puncture. Therefore, TMA with focal segmental sclerosis should first be considered based on clinical and renal pathology.

**FIGURE 3:**
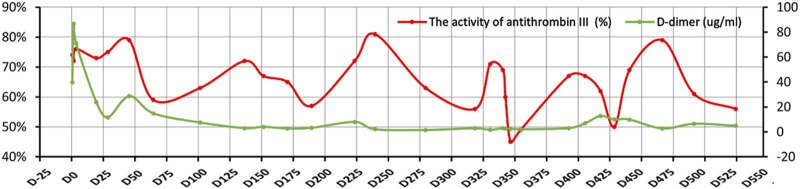
Changes in the activity of antithrombin-III and D-dimer. The *X*-axis indicates the time points of follow-up, the left *Y*-axis represents the activity of antithrombin-III, and the right *Y*-axis represents D-dimer. The red curve denotes the activity of antithrombin-III and the green curve represents D-dimer.

**FIGURE 4:**
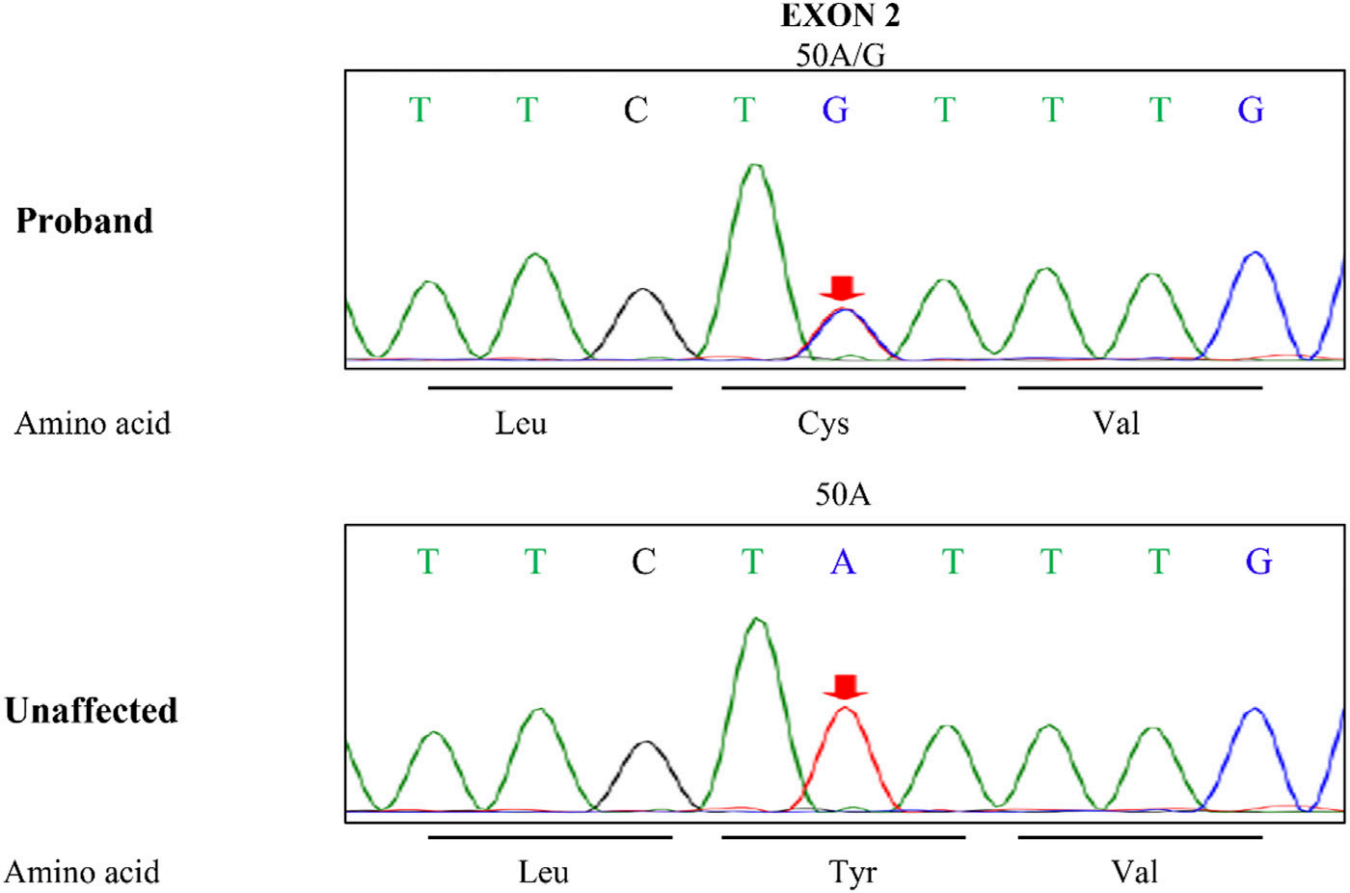
The *SERPINC1* mutation sites. Leu, leucine; Cys, cysteine; Val, valine; Tyr, tyrosine; Unaffected, unaffected member.

The activity of antithrombin III in the plasma was monitored during hospitalization by chromogenic substrate method with AT III Kit, and the results were shown below the normal range always, even below 50% (Figure 3), and the highest D-dimer value was 86.70 μg/mL. The blood mercury level was 2.9 ng/mL (reference range < 2.5 ng/mL). No specific changes were found in the blood and urine organic acid levels. Considering the patient’s multisystem thrombosis and excluding common diseases of decreased antithrombin III activity, we decided to proceed with the Next-generation sequencing (NGS)-whole exome sequencing. The captured DNA samples were taken for Illumina NovaSeq high-throughput sequencing. The sequencing data was evaluated by Illumina Sequence Control Software (SCS) for data reading and bioinformatics analysis. The result revealed that the patient had a heterozygous mutation in *SERPINC1* (NM_000488.3, OMIM ID:613118), a gene associated with thrombophilia due to antithrombin-III deficiency. The mutation site in this proband was chr1-173884049 c.50A>G in exon 2, which led to a missense mutation at the amino acid level (Figure 4). Unfortunately, information on the patient’s biological parents could not be obtained, and pedigree verification could not be performed. Consequently, TMA of the kidney was probably considered secondary to hereditary antithrombin-III deficiency. The patient was treated with enoxaparin 30 mg once every 12 h subcutaneously for anticoagulation and then switched to rivaroxaban 15 mg once daily orally because of subcutaneous hematoma. At the same time, symptomatic support treatment was given to improve renal microcirculation and protect the kidney. Unfortunately, the patient had repeated tarry stools with the lowest hemoglobin level of 28 g/L. Gastrointestinal endoscopy, as shown in Supplementary Figure S1, was successively performed and revealed erosive hemorrhagic gastritis, moderate to severe esophageal varices with a red sign, a large and tortuous rectal submucosal vascular network, and internal hemorrhoids. Patients were treated with an infusion of red blood cells and fresh plasma. During the follow-up, the patient was still hospitalized repeatedly due to gastrointestinal bleeding and anemia, and the hemoglobin level fluctuated between 30 and 50 g/L, blood creatinine fluctuated between 110 and 152 μmol/L, and D-dimer gradually decreased to a lower level; however, antithrombin-Ⅲ activity remained low. Unfortunately, the patient eventually died of severe malnutrition and gastrointestinal diseases.

## Discussion

Antithrombin deficiency can be classified as acquired or inherited. Hereditary antithrombin-III deficiency can significantly increase the risk for thrombosis. The disease is a rare autosomal dominant genetic disease caused by *SERPINC1* gene mutation, and the risk for thrombosis of different mutation types can increase up to five to 50 times (Angchaisuksiri, 2011). The disease was first reported by Egeberg in 1965 (Egeberg, 1965). The overall population incidence rate is approximately 0.02%–0.20%; however, the incidence rate in patients with thromboembolism is approximately 0.5%–5% (Lu et al., 2017). Clinically, venous thrombosis can easily occur repeatedly, which is common in limb deep vein thrombosis and pulmonary embolism; however, it can also occur in the brain or sinus, mesentery, portal, hepatic, renal, and retinal veins (Patnaik and Moll, 2008). In addition, arterial thrombosis has been reportedly associated with antithrombin deficiency (Luxembourg et al., 2014). However, TMA caused by antithrombin deficiency is rare. Our case describes a young female patient with secondary TMA associated with antithrombin-III deficiency probably caused by *SERPINC1* gene heterozygous mutation.

TMA is a common pathological manifestation in the kidney and primarily characterized by endothelial cell injury and microvascular thrombosis. Classic TMA includes HUS and TTP. Secondary TMA can be observed in malignant hypertension, tumors, autoimmune diseases, pregnancy, organ transplantation, and drug use. Although the etiology and pathogenesis of TMA are diverse, overactivation of the complement system is becoming an important mechanism (Jenny et al., 2015).

The patient had a long history of disease. At the disease onset, clinical manifestations included anemia, thrombocytopenia, and abnormal renal function. Hemolytic anemia was not observed at that time. However, renal biopsy suggested TMA. One year later, the patient presented with anemia and elevated creatinine level. Since the proportion of broken red blood cells in the patient’s peripheral blood smear was 0.1% and the platelet count was normal, there was insufficient evidence for the clinical diagnosis of TMA. Some researchers believe that renal biopsy remains the gold standard for the diagnosis of renal TMA and exclusion of other potential causes of renal injury (Brocklebank et al., 2018). Therefore, the patient was diagnosed with renal damage induced by TMA. When TMA persists and becomes chronic, multilayering of the capillary basement membrane may occur, accompanied by the absence of thrombi (Brocklebank et al., 2018). With disease progression, the clinical manifestations in this patient were not typical, which may have been related to the chronicity of the disease.

The patient had no history of relevant drug use, malignant hypertension, evidence of tumor or infection, or obvious abnormalities in autoimmune indicators, which excluded common factors that lead to secondary TMA. Additionally, the patient’s blood mercury level was slightly elevated, which was insufficient to diagnose mercury poisoning. No obvious abnormalities were found in the blood or urine organic acids, and no obvious proliferation of monoclonal plasma cells was found in the bone marrow biopsy. Therefore, TMA caused by rare factors such as methylmalonic acidemia and monoclonal gammopathy can be excluded (Morath et al., 2013; Beck et al., 2017; Yui et al., 2019). However, the activity of antithrombin-III was always lower than the normal range, even lower than 50%. Further genetic testing confirmed a heterozygous variation (c.50A>G) found in the exon region of *SERPINC1* gene. Antithrombin-III deficiency is an autosomal dominant inherited disease, which includes both homozygous and heterozygous mutations. A total of 416 mutation sites have been identified in the human gene mutation database. In this patient, a heterozygous mutation was found in the exon region of *SERPINC1*, c.50A>G, resulting in an amino acid change of p. Y17C (tyrosine > cysteine). Unlike no-sense or frameshift variants, which in most cases have a deleterious effect on the protein, missense variants have uncertain functional significance and require further studies to confirm their pathogenicity. But studies have shown that missense mutations may affect antithrombin activity by interfering with the folding of antithrombin in the endoplasmic reticulum, causing it to polymerize, further altering the conformational of serpin (Corral et al., 2018). The location of this heterotopic site was consistent with that reported in the literature, but the variation in this sample was different (Lane et al., 1997). However, the specific pathogenic mechanism of this mutation still needs further research. Unfortunately, information regarding the patient’s parents could not be obtained, and the pathogenicity could not be further verified. The patient may harbor gene mutations that have not yet been reported.

The patient had lower-limb vein thrombosis, fundus vein thrombosis, and multiple calcifications in the brain. Considering the above analysis and gene detection, the patient’s renal TMA may be caused by hereditary antithrombin-III deficiency, or the lack of antithrombin-III may be the trigger factor of TMA. To date, such cases have not been reported. However, the mechanism by which antithrombin-III deficiency leads to TMA remains unclear. Some researchers believe that patients with inherited antithrombin deficiencies develop renal failure due to fibrin deposition in the glomerulus or renal vein thrombosis (Hara and Naito, 2005). At the same time, the deficiency of antithrombin-III/*SERPINC1* can aggravate renal ischemia/reperfusion injury (Wang et al., 2015). In addition to anticoagulation, antithrombin has an important anti-inflammatory effect. It increases the production of important anti-inflammatory cytokines, such as prostacyclin, by binding with heparan sulfate on endothelial cells, thereby promoting smooth muscle relaxation, vasorelaxation, and inhibition of platelet aggregation. The lack of antithrombin can weaken anti-inflammatory effects, leading to platelet adhesion and aggregation, thrombosis, and kidney disease progression (Patnaik and Moll, 2008; Lu et al., 2017). Moreover, the patient had cirrhosis of unknown cause, secondary esophageal varices, and gastrointestinal bleeding, which may have been caused by a series of hemorrhagic diseases secondary to hepatic and intestinal microcirculation disorders due to hereditary antithrombin-III deficiency. It has been reported that antithrombin-III application could reduce both intestinal and hepatic microcirculation failure (Maksan et al., 2005), which is consistent with our conjecture. Unfortunately, antithrombin-III deficiency was detected later, and the disease progressed rapidly. The patient repeatedly experiencing gastrointestinal bleeding missed the opportunity for anticoagulation treatment, and died of recurrent gastrointestinal bleeding and severe anemia.

Gene detection was the only clue that led us to suspect that TMA was caused by hereditary antithrombin deficiency. Therefore, for patients with repeated findings of antithrombin-III activity less than 50%, the possibility of antithrombin-III deficiency and complete gene detection must be considered immediately after excluding the use of anticoagulants and lack of availability (Van Cott et al., 2020) to facilitate early detection, diagnosis, and intervention. This study has two limitations. First, we were unable to further verify the pathogenicity of antithrombin-III deficiency by sequencing genes from the patient’s biological parents. Secondly, the ADAMTS13 test was not conducted to further clarify the cause of TMA.

## Conclusion

We have reported the first case of TMA probably secondary to hereditary antithrombin-III deficiency, suggesting the importance of early identification and treatment. More cases will be needed to elucidate the pathogenesis of this disease.

## Data Availability

The datasets for this article are not publicly available due to concerns regarding participant/patient anonymity. Requests to access the datasets should be directed to the corresponding author.
