# Supplementary material for: Case report: A case of new mutation in SERPINC1 leading to thrombotic microangiopathy
Source: Front Genet. 2023 Sep 27;14:1278511. doi: 10.3389/fgene.2023.1278511 (PMC10565210; doi:10.3389/fgene.2023.1278511)
Supplement: Supplementary file 1 [file Table1.DOCX]

Supplementary Table 1. The Laboratory Values of the Patient （admission）

| Laboratory Values | Test Results | Reference Range |
| --- | --- | --- |
| WBC(10^9^/L) | 3.82 | 3.5-9.5 |
| HGB(g/L) | **62** | 115-150 |
| PLT(10^9^/L) | 177 | 125-350 |
| CRP(mg/L) | **110.50** | 0-8 |
| ESR(mm/h) | **140** | 0-20 |
| ALB(g/L) | **30.8** | 35.0-55.0 |
| BUN(mmol/L) | 7.4 | 2.30-7.80 |
| Scr(umol/L) | **119** | 44-80 |
| Cystatin C (mg/L) | **2.12** | 0.4-1.1 |
| UA(umol/L) | 323 | 150-350 |
| Glu(mmol/L) | 4.38 | 3.9-6.1 |
| DBil(umol/L) | 4.6 | 0.0-6.0 |
| IBil(umol/L) | **1.7** | 2.0-15.0 |
| LDH(U/L) | 163 | 91-245 |
| TSH(uIU/ml) | **4.350** | 0.27-4.2 |
| Anti-GBM Ab (AU/ml) | ＜2.00 | 0-20 |
| Anti-PR3 Ab(AU/ml) | ＜2.00 | 0-20 |
| Anti-MPO Ab(AU/ml) | ＜2.00 | 0-20 |
| Anti-Sm Ab(AU/ml) | ＜3.50 | 0-20 |
| ANA | Negative | Negative |
| Anti-ds DNA(IU/ml) | ＜2.00 | 0-30 |
| IgG(g/L) | **22.20** | 7.0-16.0 |
| IgA(g/L) | 3.48 | 0.7-4.0 |
| IgM(g/L) | **0.31** | 0.4-2.3 |
| C3(g/L) | 1.17 | 0.9-1.8 |
| C4(g/L) | 0.187 | 0.1-0.4 |
| HBsAg(COI) | 0.468 | 0-1.0 |
| HCVAg (S/CO) | Negative | 0-1.0 |
| TPHA (S/CO) | 0.210 | 0-1.0 |
| HIVAb (COI) | Negative | ＜0.9 |
| SG | 1.005 | 1.005-1.030 |
| UPCR(g(Tp)/g(Cr)) | **1.17** | 0-0.23 |
| UACR(g(Alb)/g(Cr)) | **0.140** | 0-0.03 |
| Ret.C(%) | **2.31** | 0.5-1.5 |
| Schistocyte(%) | **0.1** | Negative |
| Coombs’ Test  D-D(ug/ml) | **Weak positive**  **86.70** | Negative  0-0.50 |
| AT Ⅲ activity(%) | **74** | 80-130 |
|  |  |  |

WBC: White Blood Cell; HGB: Hemoglobin; PLT: Platelet ; CRP: C Reactive Protein; ESR: Erythrocyte Sedimentation Rate; ALB: Albumin; BUN: Blood Urea Nitrogen; Scr: Serum Creatinine; UA: Uric Acid; Glu: Glucose; DBil: Direct Bilirubin; IBil: Indirect Bilirubin; LDH: Lactate Dehydrogenase; TSH: Thyroid Stimulating Hormone; Anti-GBM Ab: Anti-Glomerular Basement Membrane Antiboby; Anti-PR3 Ab: Anti-Proteinase 3 Antibody; Anti-MPO Ab: Anti-Myeloperoxidase Antibody; Anti-Sm Ab: Anti-Sm Antibody; ANA: Antinuclear Antibody; Anti-ds DNA: Anti-double-stranded DNA; HBsAg: Hepatitis B Surface Antigen; HCVAg: Hepatitis C Virus Core Antigen; TPHA: Treponema Pallidum Haemagglutination Assay; HIVAb: Human Immunodeficiency Virus Antibody; SG: urine specific gravity; UPCR: Urine total protein-to-creatinine ratio; UACR: Urine microalbumin creatinine ratio; Ret.C: Reticulocyte count; Coombs’ Test: Direct anti-human globulin test; D-D: D-dimer; AT Ⅲ activity: antithrombin Ⅲ activity.

Supplementary Figure1. Gastrointestinal endoscopy shows.

**
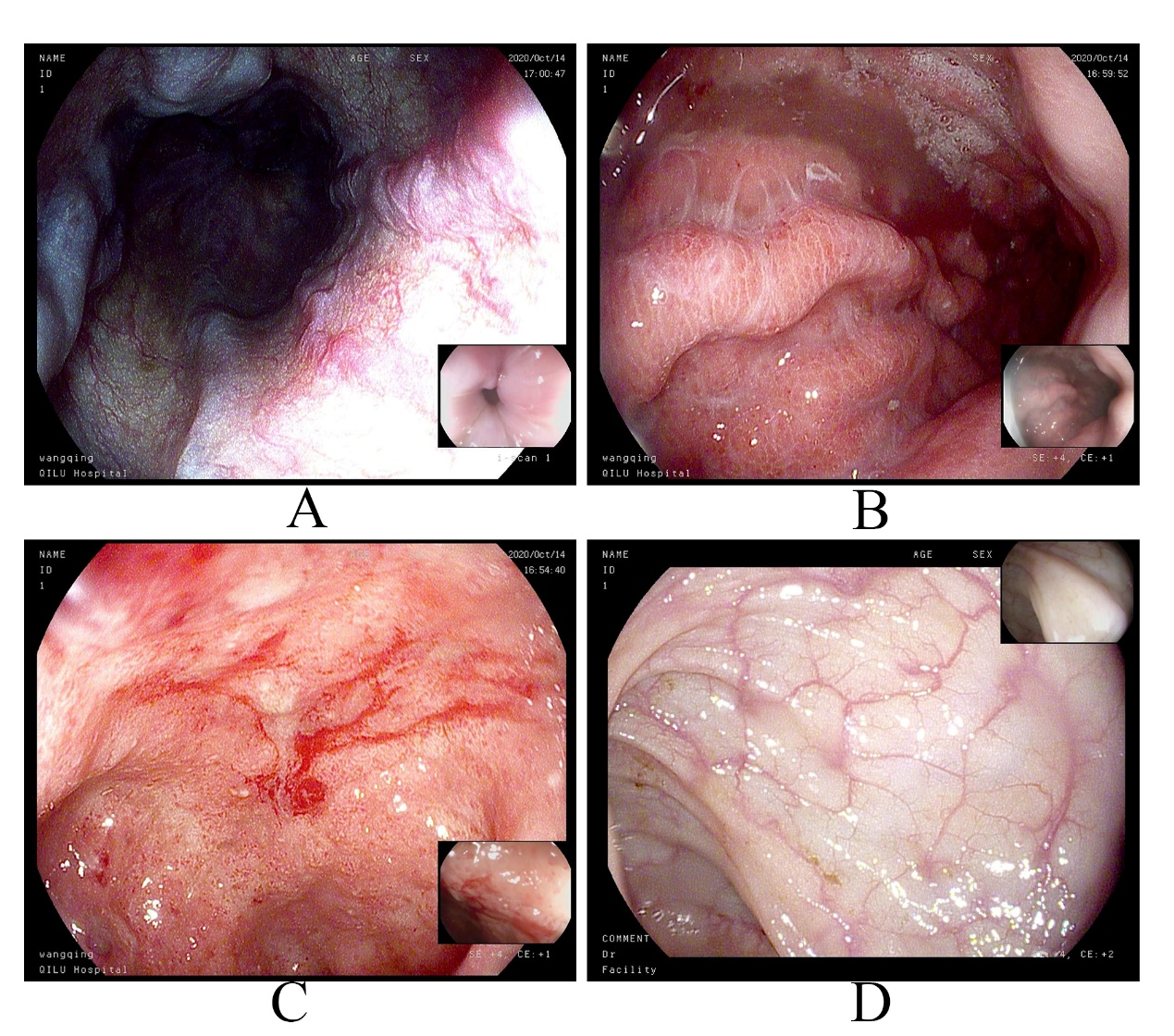
**

**(A)** The lower esophagus shows multiple varicose veins, and multiple red signs appear on the surface; **(B)** Gastric mucosa is grid- like, hyperemia edema, scattered in erosion; **(C)** The mucosa of the gastric antrum showed dense and multiple macular hyperemia spots with marginal bleeding; **(D)** The submucosal vascular network of the rectum is thick and tortuous.
